# A Systemic Review on Topical Marketed Formulations, Natural Products, and Oral Supplements to Prevent Androgenic Alopecia: A Review

**DOI:** 10.1007/s13659-020-00267-9

**Published:** 2020-10-04

**Authors:** Sumel Ashique, Navjot Kaur Sandhu, Sk. Niyamul Haque, Kartick Koley

**Affiliations:** 1 Department of Pharmaceutics, ISF College of Pharmacy, Moga, Punjab 142001 India; 2 Department of Quality Assurance and Pharmaceutical Analysis, ISF College of Pharmacy, Moga, Punjab 142001 India; 3 Department of Pharmaceutics, Gurunanak Institute of Pharmaceutical Science and Technology, Kolkata, West Bengal 700110 India

**Keywords:** Androgenetic alopecia, FDA approved drugs, Natural products, Herbal and novel topical marketed formulations, Brief descriptions about formulations, Formulation under clinical trials

## Abstract

**Abstract:**

Androgens have an intense consequence on the human scalp and body hair. Scalp hair sprouts fundamentally in awol of androgens whereas the body hair hike is vulnerable to the activity of androgens. Androgenetic alopecia (AGA) invoked as males emulate Alopecia due to the cause of the dynamic reduction of scalp hair. Androgens are medium of terminus growth of hair although the body. Local and system androgens convert the extensive terminal follicles into lesser vellus like structure. The out start of this type of alopecia is intensely irregular and the reason behind this existence of enough circulating steroidal hormones androgens and due to genetic predisposition. Effective treatments are available in the market as well as under clinical and preclinical testing. Many herbal formulations are also available but not FDA approved. Different conventional and NDDS formulations are already available in the market. To avoid various systemic side effects of both Finasteride and Minoxidil, topical formulations and natural products (nutrients, minerals, vitamins) now a days are being widely used to treat Androgenic alopecia. CAM (complementary and alternative medicine) provides the option to elect favorable, low-risk, adjuvant and alternative therapies. Herein, we offer a widespread review of topical marketed formulations, natural products, and CAM treatment options for AGA.

**Graphic Abstract:**

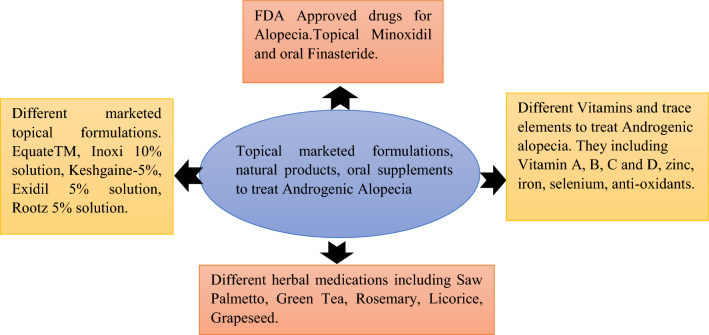

## Introduction

Two efficacious natural androgens in our body are sex steroidal hormone (5-alpha-dihydrotestosterone and testosterone). 5Alpha-reductase remains in 2 forms type 1 and type 2 and both of them mainly present in scalp follicles [1, 2]. AGA, also recognized as male or female pattern baldness affecting up to 50% of the adult male and 40% of the adult female population. Androgens bind with human androgen receptor (AR) which composed of 2 domain one is ligand bind and another is DNA bind domain. When both the steroidal hormone binds with bind site then DNA binding site activates. After the activation, the androgenic sensitive genes are exposed [3]. Androgenic receptors are normally needed in the male body for their male characterization such as testes, muscles, male reproductive systems, immune systems [4]. Studies informed that in the case of balding scalp follicles, the concentration of DHT is more intense rather than non-balding scalp follicles [5]. Experts researched and suggested that hair loss occurs due to increased concentration of both 5alpha reductase and AR, they are not surely assured about the mechanism but in hair loss, the probable reason is the gene which controls the hair follicles growth cycle is remoted by androgens [6]. Although there are only two FDA drugs (Topical Minoxidil and Oral Finasteride) are allowed for treating this AGA (Androgenic alopecia) but there are various side effects related to Minoxidil include facial hypertrichosis in 3–5% of women and contact dermatitis in 6.5% of patients and systemic finasteride also showed a large side effects including sexual dysfunction, mood disturbance and post-finasteride syndrome with related depression. At present accessible regular treatments of going bald utilizing synthetic drugs are as yet defective and have various restrictions. Their adequacy just as the security of their utilization is regularly addressed. It has prompted an expanded enthusiasm for alternative medicines with less reactions, for example, herbal plants having therapeutic potential constituents. For this herbal products now a days become crucial to treat androgenic alopecia [7]. Natural products envelop an assortment of subgroups including nutrients and minerals, botanicals, and probiotics, which are all internationally showcased as dietary enhancements and don’t need Food and Drug Administration (FDA) endorsement. The 2012 National Health Interview Survey reports concluded that CAM (complementary and alternative medicine) approach for dermatological conditions were utilized by 17.7% of Americans [8]. The reports showed the enhanced hair growth by using amino acids, caffeine, capsaicin, curcumin, garlic gel, marine proteins, melatonin, onion juice, procyanidin, pumpkin seed oil, rosemary oil, saw palmetto, vitamin B7 (biotin), vitamin D, vitamin E, and zinc.

## Management of Androgenic Alopecia

Beyond treatment, the androgenic alopecia increasing day by day. Researchers found that approximately the rate is near about 5% per year. There are many disguises and surgical management procedures are available but, in this paper, we have discussed the therapeutic management procedures. Figuring out the functional sequence alternate in or around the AR gene will lead to the dedication of the exact variation in AR proteins between bald and non-bald people. By this proficiency, treatments can be arranged that the point and reverse these inequalities, through that impeding exact hair loss mechanism. Now recent pharmaceutical treatments for androgenic alopecia do not mark the particular cellular mechanisms in this procedure. Rather they impede the activity of the enzyme which boosts the AR in the balding scalp, thus they are abolishable than curative, with different success rates.

### FDA Approved Drug for Alopecia

**Fig. 1:**
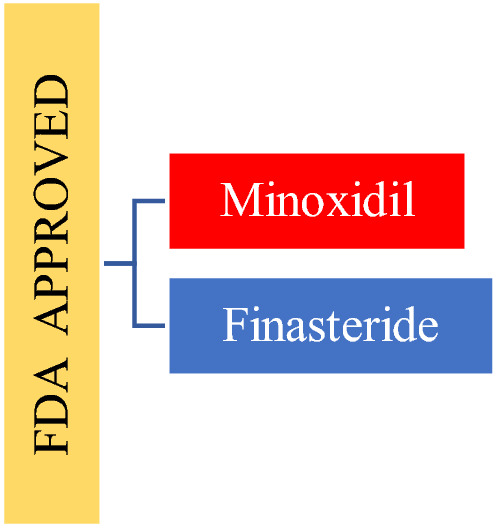
FDA approved drugs

**Fig. 2:**
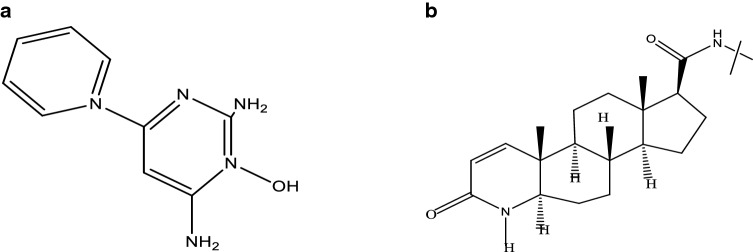
**a** Chemical structure of Minoxidil. **b** Chemical structure of Finasteride

Drugs that are approved by the FDA for the treatment of alopecia are shown in Fig. 1 and their respective chemical structures are in Fig. 2a and b.

Only two approved drugs by the FDA still are there for Alopecia are Finasteride and Minoxidil. The patent of Finasteride was filed in 1984 and accepted for medical purposes in 1982 and was available in generic form. Whereas Minoxidil was developed in the 1950s by the Upjohn Company (now as Pfizer). Then the company had synthesized many derivatives and in 1963 named Minoxidil [9]. In 1979 it was authorized by FDA for the treatment of high BP in tablet form with Loniten trade name [10]. In 1988 FDA approved it for treating male pattern baldness in men with the trade name of ‘Rogaine’ [11]. In 1998, 5% of Minoxidil formulation was allowed by the FDA [10]. In the year 1998 minoxidil came for sale nonprescription ally by FDA and in 2014 it was the only topical choice by FDA approved for treating androgenic alopecia [12]. The drug is available in the topical formulation in the UK, US, Sweden, and Germany.

#### Minoxidil

At first, Minoxidil was used to treat high BP due to its systemic side effects. The formulation changed to the topical formulation used to treat baldness [13]. Minoxidil is present as a 2%, 5% topical solution approved by USFDA. In 1998 it was approved first for male pattern baldness and then for female in 2001 as 2% minoxidil solution and 5% minoxidil was approved in 2007 for male androgenic alopecia. 5% Foam minoxidil also approved by FDA in 2006 but only in men, not in case of women’s hair loss purpose, it was off-label treatment formulation. 2% and 5% Topical solution indicated twice a day as 1 mL of the solution.

#### Finasteride

The 5α-reductase inhibitor finasteride blocks the conversion of testosterone to dihydrotestosterone (DHT), the androgen responsible for male pattern hair loss (androgenic alopecia) in genetically predisposed men. Finasteride, another FDA approved drug has been reported effective in 0.25% and 0.5% topical solution compared to an oral 1 mg/dose. Oral Finasteride tablet have different systemic side effects, to overcome these problems topical formulation of Finasteride now-a-days successfully applied on male pattern alopecia.

**Fig. 3:**
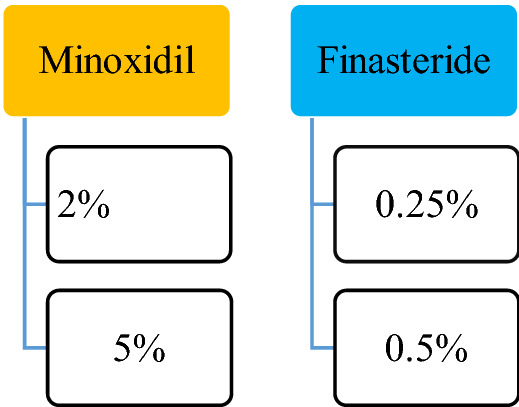
% drug effectiveness of FDA approved drugs

% Drug effectiveness [14, 15] for the FDA are elaborated in Fig. 3.

### Adverse Effects of Both FDA Approved Drug Formulation

Common side effects or adverse effects for both the drugs i.e. Minoxidil and Finasteride are illustrated in Table 1.

**Table 1:** Side effects of minoxidil and finasteride

Side effects of Minoxidil	Side effects of Finasteride	References
Dryness of scalp	Impotence	[16]
Irritation on skin	Decreased libido	[17]
Rashes	Erectile dysfunction and testicular pain	[18]
Burning	Ejaculation disorders	[16]
Redness and dermatitis	Headache	[17]
Drying of skin	Dizziness	[18]

## Role of Various Oral Supplements Like Vitamins and Minerals to Prevent Androgenic Alopecia

Dietary micronutrients such as vitamins, minerals are nowadays becoming another option for treating androgenic alopecia. These minerals play a key role in the normal hair follicle cycle. The deficit of micronutrients represents an adjustable risk factor associated with the development, prevention, and treatment of alopecia. Vitamins and minerals are vital for normal cell growth and function and may cause hair loss due to deficiency of them [19]. Where supplementation is relatively inexpensive and easily available, now it will be discussed which vitamins and minerals help treat hair loss. In total hair follicles, about 90% of them are present in the anagen phase where there is no chance of hair loss. Some essential elements, such as proteins, vitamins, and minerals are very much needed to produce healthy hair [20]. So, micronutrients, including vitamins and trace minerals, are therefore vital components of our diet [21] (Table 2).

**Table 2:** Brief elaboration of different vitamins to treat androgenic alopecia

Vitamin A	Keeping homeostasis and by delay, the proper concentration of active metabolite is more important for healthy hair [22] but consuming very much or over-supplementing vitamin A can cause hair loss [23]. One study concluded that the mouse AA model, reduction of vitamin A in the diet truly hindered hair loss onset [24]. Vitamin A deficiency results in ichthyosis-like skin changes and sometimes causes telogen effluvium and fragility of the hair. In vitamin, A deficiency, a single dose of 200,000 IU is given by mouth every 4–6 months
Vitamin B	The vitamin B complex contains eight water-soluble vitamin substances thiamine (B1), riboflavin (B2), niacin (B3), pantothenic acid (B5), vitamin B6, biotin (B7), folate, and vitamin B12. Among them, only riboflavin, biotin, folate, and vitamin B12 deficiencies have been associated with hair loss. Fujimoto et al. reported lacking sufficient biotin content resulted in periorificial dermatitis and patchy alopecia, both of which resolved with daily oral supplementation of biotin [25]. Biotin deficiency can cause hair loss, skin rashes, and brittle nails, the efficacy of biotin in supplements for hair, skin but not applicable in large-scale studies. Only case reports have been used to justify the use of biotin supplements for hair growth [26]. Folate and vitamin B12 in nucleic acid production concluded that they play a key role in highly proliferative hair follicles [27]. Logihair Soft Gelatin Capsule is a marketed formulation for treating hair loss and it contains B6 and Folic acid Biotin, Choline, Elemental Selenium, Elemental Zinc, Folic Acid, Niacinamide, Saw Palmetto, and Vitamin B6
Vitamin C	Vitamin C plays a vital role in the intestinal absorption of iron having its chelating and reducing effect. Therefore, vitamin C oral supplements in patients with hair loss associated with iron deficiency. Vitamin C endorses hair shaft elongation in cultured human hair follicles and prompts hair growth in mice Through the progression from telogen to anagen. This was achieved by enhancing the Insulin Growth Factor 1 (IGF1) production in the dermal papilla cells. The recommended treatment for Vitamin C deficiency is 300–1000 mg daily of oral vitamin C for 1 month [28]
Vitamin D	Vitamin D in the hair follicle is evinced by hair loss in patients with vitamin D-dependent rickets type II. Patients having mutations in the VDR gene, resulted in vitamin D resistance and sparse body hair, repetitively linking with total scalp and body alopecia. VDR (vitamin D receptor) spreading on the body is not limited to organs but also present in the immune system. In the hair follicle, the VDR is expressed in the mesodermal dermal papilla cells and the epidermal keratinocytes depending on the stage of the hair cycle. VDR is overexpressed in a hair follicle during late anagen and catagen, correlating with proliferation and differentiation of the keratinocytes in producing for the new hair cycle [29]. Deficiency of VDR in the keratinocytes as opposed to the dermal papilla results initiating of subsequent anagen phase [30]. Therefore, VDR provides a key role in the hair cycle, independent of the vitamin D binding. D2 (ergocalciferol) and D3 (cholecalciferol) are available as dietary supplements. Vitamin D topical analogs have been tested in mice with congenital alopecia with a positive response [31]. A one-time dose of vitamin D3 of at least 300,000 IU is most effective in improving vitamin D status for up of 3 months
Vitamin E	A case study reported minimal information concerning the benefits of vitamin E supplementation on hair loss. A study of 21 volunteers who received tocotrienol supplementation (100 mg of mixed tocotrienols daily) exhibited a significant increase in hair number as compared to a placebo group [32]. Vitamin E has antioxidant properties that help to reduce oxidative stress in the scalp. Oxidative stress has been linked with hair loss

### Role of Essential Trace Elements in Hair Loss (Table 3)

**Table 3:** Trace elements for androgenic alopecia

Iron	Iron deficiency, which contributes to androgenetic alopecia (AGA), telogen effluvium [33]. Iron deficiency is mainly linked with women pattern hair loss [34]. Trost et al. [35] and Pierre et al. [36] has been reviewed about the deficiency of iron in male pattern baldness [37]. The recommended oral daily dose for the treatment of iron deficiency in adults is in the range of 150–200 mg/day of elemental iron
Zinc	Alopecia is an eminent sign of established zinc deficiency with hair regrowth occurring with zinc supplementation [38]. One case report with a patient having dry brittle hair and alopecia due to zinc deficiency but the improvement was seen in alopecia following oral zinc therapy [39]. Kil et al. [40] described the correlation between hair loss and deficiency of zing oral supplements in the case of telogen effluvium and male pattern hair loss. Zinc acts as a hair growth modulator and immunomodulator as DNA polymerase is zinc-dependent and zinc acts in multiple aspects of T-lymphocyte activation, signal transduction, and cellular apoptosis [41]. Zinc sulphate in a dose of 5 mg/kg/day for 3 months in patients with alopecia areata
Selenium	This supplement plays a key role to prevent oxidative damage and also hair follicle morphogenesis. Bates et al. [42] reported that selenium supplements improved scattered hair growth. The recommended daily intake dose is 25–50 mcg. Hairgro Forte Tablet is a marketed formulation for treating hair loss. It contains Folic Acid, Biotin, Iron, Calcium Pantothenate, Acetylcysteine, Zinc, and Copper
Antioxidants	Antioxidants play a key role to neutralize reactive oxygen species (ROS), which leads to preventing oxidative damage. Many substances can be classified as antioxidants, including zinc, selenium, and vitamins A and E. Oxidative stress has been linked to hair loss. In vitro studies of dermal papilla cells from male AGA, patients concluded that oxidative stress plays a role in the balding phenotype and development of androgenetic alopecia [43]

## Role of Dandruff in Hair Fall

Dandruff is a common scalp disorder, characterized by the presence of corneocytes that form clusters due to their high cohesive power, in the form of flaky white to yellowish scales, accompanied by itching and mainly occurs when sebaceous glands are most active.

Dandruff directly not responsible for hair loss, but the two may be linked indirectly. Due to some infections and medical conditions are responsible for dandruff and hair loss. The harshness of dandruff plays a role in severe conditions among the subjects; the scales may be trapped in a crowded terminal. The presence of dandruff may lead to telogen effluvium [44] and may also cause Androgenic alopecia. However due to dandruff itchiness occurs which leads to scratching and injure the hair follicles, leading to hair loss to some extent. One common dandruff-related health problems that may cause hair loss is seborrheic dermatitis. Seborrheic dermatitis is a flaky, scaly rash that forms on the scalp and face. When it develops on the scalp, it causes dandruff and creates temporary hair loss. New York dermatologist Michele Green, MD says FDA approved drug Minoxidil can cause dandruff-like flaking as a side effect. The presence of alcohol in minoxidil can dry out the scalp, and after a few months of treatment, dandruff may develop, and on the other hand, Finasteride, another medication for hair loss, does not cause flaking.

## Role of PRP (Platelet-Rich Plasma) in Hair Regrowth

Platelet-rich plasma therapy has become a new popular treatment for hair regrowth in male pattern baldness. Activated platelets release numerous growth factors and cytokines from their alpha granules as part of the wound healing process. Platelets in PRP become activated when injected into the scalp and release multiple growth factors, which promote hair growth. Khatu et al. [45] reported 11 patients who were suffering from Androgenic Alopecia taking Finasteride and Minoxidil for 6 months but there was no improvement in hair growth. A total volume of 2–3 cm^3^ PRP was injected in the scalp with an insulin syringe. The treatment was done for every 2 weeks, for a total of four times. The result was assessed after 3 months through clinical examination, macroscopic photos, hair pull test, and patient’s overall satisfaction. The result showed that hair count improved from an average number of 71 hair follicular units to 93 hair follicular units. That means the average mean gain was 22.09 follicular units/cm^2^. After the fourth session, the pull test was negative in 9 patients among 11. A crucial decrease in hair loss was observed between the first and fourth injection. Gkini et al. [46] did a nonrandomized trial to check the efficacy of PRP injection in 22 patients affected by androgenic alopecia. This study based on 3 treatment sessions with an interval of 3 weeks. At 6 months from the starting of the treatment, a booster assembly was also performed and hair density significantly increased at 6 weeks. Kang et al. [47] reported the clinical efficacy of injection of CD34+ cell-containing PRP preparations for male and female pattern hair loss. In this study, 3 months after the first treatment, the results showed clinical improvement in the mean number of hairs (20.5% ± 17.0%), mean hair thickness (31.3% ± 30.1%). At 6 months, the results were increased in mean hair count (29.2% ± 17.8%), mean hair thickness (46.4% ± 37.5%). Trink et al. [48] reported the effect and safety of PRP on alopecia areata in a randomized, double-blind, placebo and active-controlled, half-head, parallel-group study. 45 Patients having alopecia areata were given intralesional injections of PRP and triamcinolone acetonide (TrA). Gentile and Garcovich [49] described a systematic review of Platelet-Rich Plasma Use in Androgenetic Alopecia Compared with Minoxidil, Finasteride, and Adult Stem Cell-Based Therapy. The results showed patients who were treated with PRP had significantly increased hair regrowth compared with those treated with TrA. Platelet-rich plasma (PRP) has appeared as a new treatment modality in reformative plastic surgery, and the preliminary result suggested that it might have a valuable role in hair regrowth. The results of a randomized, placebo-controlled, half-head group study to compare the hair regrowth with PRP versus placebo are reported and it was seen that in every aspect of view PRP showed better results than other options. PRP tends to progress hair caliber and hair growth for about 4 to 6 weeks which needed repeated treatments once a month for 3 months. On average most patients must require repetitive PRP treatment after 6–12 months to keep the hair growth effects. In the case of hair loss, the injected platelets prompt inactive or newly implanted hair follicles to enter an active growth phase, causing the hair to start growing again.

## Role of Low-Level-Laser Therapy Hair Comb in Hair Loss Prevention

Nowadays laser comb has been approved by the FDA for both safety and effectiveness in case of the treatment of the hair loss. It is a non-chemical, non-invasive option to help people grow fuller, thicker, healthier hair [50]. LLLT stimulated hair growth in mice when they have induced chemotherapy for producing alopecia and also in alopecia areata. Various controlled clinical trials confirmed that LLLT stimulated hair growth in both men and women. There are many mechanisms are established but among them, the key mechanism is the stimulation of epidermal stem cells in the hair follicle bulge and shifting the follicles into anagen phase which promotes hair growth as well as prevents hair loss [51]. In the late 1960s, Endre Mester, a Hungarian physician experimented on the carcinogenic potential of lasers by using a low-power ruby laser (694 nm) on mice. It was seen that the laser did not cause cancer and unexpectedly improved hair growth around the shaved region on the animal’s back [52]. Laser phototherapy is supposed to stimulate anagen phase re-entry in telogen phase hair follicles that extend the duration of the anagen phase, enhances rates of proliferation in active anagen hair follicles, and to avoid premature catagen development [53]. LLLT has been demonstrated to modulate inflammatory processes and immunological responses, that result in an effect in hair regrowth [54]. Wikramanayake et al. described the C3H/HeJ mouse model which was treated with laser comb and resulted in an enhanced number of hair follicles with the majority in the anagen phase were observed with reduced inflammatory infiltrates. Shukla et al. [55] studied the outcome of helium–neon (He–Ne) laser on the hair follicle growth cycle of testosterone-treated and un-treated Swiss albino mice skin and the result showed testosterone treatment led to the inhibition of hair growth that was characterized by substantial growth in catagen follicles. The results showed that testosterone-treated mice to the He–Ne laser led more hair follicles in the anagen phase when compared to the other groups. Satino and Markou [56] tested the efficacy of LLLT on hair growth and tensile strength on 28 male and 7 female androgenic alopecia patients who were given a HairMax LaserComb® 655 nm, to use at home for 6 months for 5–10 min every other day. Results show in the case of hair tensile strength it was seen that better improvement in the vertex area for males and temporal areas for females but both sexes got benefited in all areas ominously. LLLT reported very few side effects over the past 50 years. LLLT has only one adverse effect in humans was the temporary onset of TE developing in the first 1–2 months after instigation LaserComb treatment. However, more studies are required to optimize treatment parameters and determine long-term efficacy as well as the safety of emerging LLLT technologies.

## CAM Therapy to Treat AGA

Though there are a variability in CAM treatment choices on the market for both androgenic alopecia and alopecia areata, only a few completed multiple randomized controlled clinical trials. So, there is a requirement for additional studies about CAM for alopecia with more robust, clinical design and standardized, quantitative results.

### Acupuncture

The concepts behind this treatment are it can enhance blood circulation, stimulate the hair follicles and diminish inflammatory infiltrates [57]. It was reported that 78 patients were treated with plum-blossom acupuncture and with 2% topical MXD. Results showed total regrowth was 58.1% in acupuncture therapy whereas 34.3% in MXD treated patients [58]. A case study reported improvement in AGA by combining therapy of pharmacopuncture, acupuncture and needle [59].

### Aromatherapy

This therapy uses massaging of essential oils with jojoba, grape seed carrier oils on scalp. Reports said that eighty four patients were treated for 7 months and result showed that 44% patients having increased hair growth whereas only 15% who were treated with carrier oil only [60].

### Psychotherapy

This treatment directly works on the state of mind. A report said that treatment with additional relaxation and prednisolone 5–10 mg/day for 30 min, 2 months and further prednisolone for another 4–5 months improved hair growth in 83% of treated people.

## Natural Sources for Medication

Androgenetic Alopecia can be treated with the help of some natural marketed preparations. These natural preparations commonly employed due to their less or no toxic effects means these all preparations are safe to use. Some of the medications whose natural sources are elaborated in Table 4 where their Composition, Action, and Dose are well described.

**Table 4:** Different natural sources medication related to androgenetic alopecia

S. Nos	Sources	Composition	Mechanism of action	Dose	References
(1)	Saw Palmetto	Contains fatty acids (85–90%), carotenoids, lipases, tannin, sugars and beta-sitosterol, anthranilic acid, capric acid, caproic acid, caprylic acid, carotene, ferulic acid, linoleic acid, myristic acid, lauric acid, oleic acid, palmitic acid, 1-monolaurin, and 1-monomyristin	Existing ingredients show inhibition of 5-alpha-reductase out of which lauric acid, myristic acid, and oleic acid may be the main fatty acids responsible	320 mg/day	[61–63]
(2)	Green tea	Antioxidants such as polyphenols and flavonoids that contains catechins and its derivatives epicatechin (EC), epigallocatechin gallate (EGCG), epigallo catechins, and epicatechin gallate, linoleic and linolenic acids, vitamins, etc	EGCG is the main component of green tea that stimulates human hair growth via its proliferative and antiapoptotic function on dermal papilla cells also affects type I, 5α reductase activity that converts testosterone to DHT		[64–68]
(3)	Pumpkin seed	Presents polyunsaturated fatty acids of 80% palmitic acid, myristic acid, stearic acid, oleic acid, and linoleic acid, vitamin E like α-tocopherols, γ-tocopherols and carotenoid, phytoestrogens, and phytosterols and trace components	Inhibit 5-alpha-reductase activity	400 mg/day for 24 weeks	[69–71]
(4)	Rosemary	It contains esters (2.6%) largely as borneol, cineoles, and several terpenes, chiefly a-pinene, camphene, 1%, 2% volatile oil containing 0.8%, 6% of esters and 8%, 20% of alcohols	Acts by improving blood circulation and improving vascularity helping the regeneration of follicles similar effect that is shown by minoxidil		[72]
(5)	Grape seed	Anthocyanins, flavan-3-ols (example: catechins), vitamin-E (α-tocopherol), petiole, linoleic acid, flavonoids (resveratrol, quercetin and catechin, and polyphenols (flavonoids, phenolic acids, phenolic alcohols, stilbenes, and lignans), and trimer gallate, unsaturated fatty acids, and phytosterols			[73]
(6)	Licorice	Glycyrrhetinic acids, rich in flavonoids such as liquiritin, isoliquiritin, neoisoliquiritin, liquiritigenin, glyzarin, glyzaglabrin, licoisoflavones	Presence of glycosides, terpenoid, phenolics, and flavonoids are widely available having antagonizing testosterone effect		[74, 75]

### Different Natural Products for Preventing Hair Loss

According to the National Center for Complementary and Integrative Health (NCCIH), a branch of the National Institutes of Health (NIH, Bethesda, MD, USA) has approached for natural products. CAM (complementary and alternative medicine) offers to select promising, low-risk, adjuvant, and alternative therapies. Here a comprehensive update of CAM treatment options for alopecia, with most evidence in androgenetic alopecia (AGA) has discussed. Natural products are categorized in various subgroups including vitamins and minerals, herbs/botanicals, and probiotics, all of which are globally marketed as dietary supplements and do not require the Food and Drug Administration (FDA) approval (Table 5).

**Table 5:** Natural methods for preventing hair loss

Onion juice	Though the exact mechanism of action towards hair growth is unknown this natural product is quite popular for preventing hair loss purposes. The only patient compliance is the unpleasant odor of it. Presence of sulfur and phenolic compounds are responsible for hair growth purpose. Topical crude onion juice was applied in 62 patients and after 8 weeks it was reported that improvement was seen in the patients [76]
Rosemary oil	Rosemary (*Rosmarinus officinalis* L.) herb has different useful properties such as antioxidant, antibacterial, antifungal, and anti-inflammatory. It enhances microcapillary perfusion which improves hair growth. A comparative study was carried among 100 androgenetic alopecia patients with topical rosemary oil lotion (3.7 mg/mL) applied daily and topical 2% minoxidil. A standardized professional microphotographic assessment of each volunteer was taken at the initial interview and after 3 and 6 months of the trial. It was seen in both groups it enhanced the hair count after 6 months and the only common adverse effect reported was scalp itching, more frequent with Minoxidil use [77]
Saw Palmetto	SP (Saw Palmetto) is a competitive, nonselective inhibitor of both forms of 5α-reductase. SP blocks nuclear uptake of DHT in target cells and decreases DHT binding to androgen receptors by approximately 50%. The SP extract proliferates 3α-hydroxysteroid-dehydrogenase activity and enhances the conversion of DHT to its weaker metabolite. Prager et al. [78] tested on 26 androgenetic alopecia patients treated with either 50 mg of oral β-sitosterol and 200 mg SP and it was reported 60% improvement in hair loss but has some side effects including appetite, flatulence, and diarrhoea. Another study was carried out with SP as a topical agent. Evaluating the hair growth effect of 3.3 mL topical SP serum applied for 4 weeks and 2 mL lotion for 24 weeks, in 50 men having AGA and reported increased average and terminal hair counts at 12 and 24 weeks [79]
Pumpkin seed oil	Pumpkin seed oil (PSO) contains phytosterols which are a 5α-reductase inhibitor that prevents the conversion of testosterone to active dihydrotestosterone (DHT) and thus improves the hair growth [80]. A comparative study of 400 mg of oral PSO daily to placebo for 24 weeks in 76 patients with AGA verified enhanced in hair count of 40 versus 10% with placebo. PSO is a promising treatment for AGA involving the vertex but failed on frontal variants
Procyanidin	Topical 1% procyanidin B2, derived from apple juice, reported significant improvement in total and terminal hair counts at 4 months and 6 months in 29 patients having male pattern alopecia compared to placebo [81]. Procyanidin 0.7% used to treat 43 men with AGA also established enhanced hair growth counts (3.3 vs. − 3.6 for placebo) after 6 months, with a total increase of 23 hairs/cm^2^ after 12 months [82]
Garlic gel	A trial was conducted of 40 alopecia patients with topical 5% garlic gel in combination with betamethasone was evaluated in comparison to placebo. After 3 months a positive response was observed in 95% of those treated compared to 5% with placebo [83]
Capsaicin	Oral capsaicin 6 mg and isoflavone 75 mg daily for 5 months improved serum IGF-I in patients with AGA. In AGA specifically, 88% observed hair growth was seen in that treatment [84]. Another research showed that topical 0.01% raspberry ketone (structure similar to capsaicin) regulates IGF-I and stimulates hair growth in 50% of patients [85]
Caffeine	Caffeine enhances cellular proliferation, counteracts the inhibitory effects of testosterone on hair growth, promotes hair shaft elongation, extends anagen duration, and stimulates hair matrix keratinocyte proliferation [86]
Amino acids	Oral l-cystine (70 mg) in combination with retinol was assessed for the treatment of diffuse alopecia and resulted in enhanced hair density and anagen rate [87]. Oral l-cystine was also trailed in combination with histidine, copper, and zinc is taken 4 times daily which results in improved mean change in total hair count after 50 weeks) in 24 patients having AGA [88]
Curcumin	A 5% topical hexane extract of *Curcuma aeruginosa* (CA) was compared to placebo, 5% minoxidil. On photographic review, combination therapy and 5% minoxidil showed significant improvement than single treatment while the subjective valuation of hair regrowth was only ominously enhanced in the combination group

### Herbal Medication for Treating Alopecia and Their Marketed Herbal Formulations

Herbal formulations are nowadays has become more eye-catching due to some specific advantages over a novel drug delivery system and dosage form. Origin of herbal formulations is mainly natural occurring, therefore; it provides fewer side effects and toxicity than the synthetic carrier for alopecia. Some of the Herbal Formulations which are available in the market are described in detail.

#### Saw Palmetto

The market herbal formulation for Saw Palmetto [89] is given in Table 6 which describes the source, type of dosage form, the dose that can be given, route of administration, name of a brand for which formulation is available in the market, usage about the formulation and cautions that one kept in mind before using the formulation. Not only this their storage (as is the important part of any formulation whether that formulation is prepared from natural or synthetic forms), mode of action, possible side effects, and their interaction also mentioned.

**Table 6:** Market formulation for Saw Palmetto

Herbal source	Dosage form	Dose	Administration route	Brand name	Direction for use	Caution
Saw palmetto (OTC drug)	Saw palmetto berries, capsule	500 mg	Oral	Daily wellness	1 Capsule daily with a meal	Pregnant or nursing mothers, children under 18 years old
	Formula 82S-shampoo and conditioner		Topical	Simply herbal	16 OZ	Keep away from children
	Saw palmetto and cayenne		Topical	Art naturals	4 FL OZ	
				Botanical		
Storage	Mode of action	Side effects	Interaction			
Keep in a dry place	prevents testosterone from being converted to a more potent form of testosterone (DHT)	dizziness, headache, nausea, vomiting, constipation, diarrhea	Along with estrogen pills might decrease the effectiveness of estrogen			

#### Green Tea

Green tea has beneficial effects such as anti-cancer and anti-oxidant properties mediated by epigallocatechin-3-gallate (EGCG), a major constituent of polyphenols. Recently, reported that EGCG can be useful in the treatment of androgenetic alopecia by selectively inhibiting 5alpha-reductase activity [90]. The Market formulation for green tea its dosage form, Route for application and how to use given in Table 7.

**Table 7:** Market formulation for green tea

Herbal source	Dosage form	Route	Direction for use
Green tea	Centella 65 Green Tea Pack, Cream	Topical	Apply gently on the scalp
	Medi Sun Soothing, Cream	Topical	Gently on the scalp by finger
	Anti-hair loss cream, 100 mL, 50 mL	Topical	8 OZ. Rinse gently allows for 2–3 min
	Hair loss shampoo	Topical	

#### Rosemary

Rosemary is a sweet-smelling evergreen herb that has medicinal properties. Conventionally it is used to improve memory, reduces muscle pain, speeds up the immune and circulatory system, and help in hair growth. Rosemary has potential health benefits which include antioxidant and anti-inflammatory compounds, helps in improving digestion, memory enhancement, increasing concentration, helps in neurological protection, helps in preventing brain aging, reduces the formation of cancer-causing agents that acts as an anti-tumor agent. Several side effects are generated due to an increase in the dose. Side effects include coma, muscle spasms, vomiting, and an increase in dose lead to miscarriage; therefore, pregnant women should avoid the use of any rosemary products [91].

A brief about the rosemary market formulation [92] given in Table 8 which includes its dosage form, brand name, the dose that can be taken, route, and how to apply.

**Table 8:** Market formulation for rosemary

Herbal source	Dosage form	Brand name	Dose and route	Direction for use
Rosemary	Oil	Soulflower.biz	4–5 drops with 1 teaspoon coconut oil, topically	Apply onto hair and scalp

#### Grapeseed

Grapeseed, a byproduct of the wine production from grapes. Other names of grape seed are Grape-seed oil, Grape seed extract, and many more. Grapefruit juice or Grapefruit products are different from grape seeds. As researchers had said that grape seed is not effective in the treatment of allergies related to seasonal changes. Several side effects of grape seed include vomiting, nausea, dry mouth, cough, headache, muscle pain, and upset stomach. There are some drugs with which grape seed cannot be administered includes; some antidepressants; asthma medicines; medicines related to heart or blood pressure; medicines for the treatment of mental illness or anxiety [93].

Description of the market formulation of grape seed [94] given in Table 9 which has dosage form, name of brand available in market, route, and direction for use.

**Table 9:** Market formulation for grape seed

Herbal source	Dosage form	Brand name	Route	Direction for use
Grape seed	Oil	PRZ Herbals Care	Topical	0.5 fl. OZ massage (skin and hair)

#### Licorice

Licorice an herb that belongs to the Mediterranean, southern, and central Russia. Some of the species nowadays grown over Europe, Asia, and the Middle East. Licorice contains the acid i.e. Glycyrrhizic acid that can cause problems when consumed in larger quantities. Licorice can be administered in a single form or can be combined with other herbs like problems related to the digestive system; ulcers, heartburn, chronic gastritis. In several conditions like itchy skin, inflamed skin, psoriasis, or brown spots licorice applied to the skin. Licorice also used in foods for flavoring, in beverages, and tobacco products [95].

Brief about the Licorice available market preparation [96] given in Table 10.

**Table 10:** Market formulation for Licorice

Herbal source	Dosage form	Brand name	Route	Direction for use
Licorice	Shampoo	Soultree	Topical	250 mL massage to wet hair

## Marketed Topical Formulation for Androgenic Alopecia

### Minoxidil Topical Solution USP 5% (w/v)

Mintop Forte 5% Solution is a medicinal drug used in the cure of male pattern baldness. It is the highest quality in treating familial hair loss or thinning at the top of the scalp, no longer in front. Mintop Forte 5% Solution helps to stimulate hair increase with the aid of growing the blood circulation to the hair follicles. However, the amount of hair growth is one-of-a-kind for every person. During the first 2 weeks of application, your hair fall may extend temporarily. This is every day and is a sign that the remedy is working. This medicine must strictly be taken following the doctor’s advice in Table 11.

**Table 11:** Minoxidil topical solution USP 5% (w/v)

Manufacturer	Composition	Types	Volume	Brand name	Gender
Dr. Reddy’s Laboratories Ltd., India	Minoxidil 5% (w/v)	Topical solution	120 mL in 1 bottle	Mintop ForteTM, Here restore Formula	For male

#### Mechanism of Action

Minoxidil is a vasodilator it widens the blood vessels which improves blood flow. Using Mintop solution on the top of scalp increase the blood flow which provides more the oxygen and nutrient to the hair follicle that lower the death of hair follicle cell. This medication triggered the anagen chemical messenger.

#### Expert Comments

Should be applied directly to the scalp area. If it comes in contact with eyes and mouth immediately wash with cool water properly. Do not use a hairdryer as it reduces effectiveness. Do not apply shampoo for 4 h after applying the Mintop Forte solution. Use carefully because in contact with face it will cause unwanted facial hair growth.

Some of the alternative brands for Mintop Forte Solution have been described in Table 12 with their; brand name, the composition of salt, type of dosage form, name of the manufacturer, and the volume of the dosage form.

**Table 12:** Alternative brands for Mintop Forte solution

Brand	Salt Composition	Dosage form	Manufacturer	Volume (mL)
Tugain	5% Minoxidil	Topical Solution	Cipla Ltd.	60
Regaine	5% minoxidil	Topical Solution	Janssen Pharmaceuticals	60
MX	5% Minoxidil	Topical Solution	Hedge and Hedge	60
Minoqilib	5% Minoxidil	Topical Solution	Galderma India Pvt Ltd.	60
Coverit	5% Minoxidil	Topical Solution	Micro Labs Ltd.	60
Morr	5% Minoxidil	Topical Solution	Intas Pharmaceuticals Ltd.	60
Checkfall	5% Minoxidil	Topical Solution	Mankind Pharma Ltd.	60
Minicheck	5% Minoxidil	Topical Solution	Abbott	60
Imxia	5% Minoxidil	Topical Solution	KLM Laboratories Pvt. Ltd.	60
Rootz-M5	5% Minoxidil	Topical Solution	Apple Therapeutics Pvt. Ltd.	60
Black Crown Forte	5% Minoxidil	Topical Solution	Derma Joint India	60
Gainehair	5% Minoxidil	Spray	Wockhardt Ltd.	60
Vivadil Forte	5% Minoxidil	Solution	Biochemix Health Care Pvt. Ltd.	60
Recapil PM Solution	5% Minoxidil	Solution	Mascon Health Care Ltd.	60
Kera XL M Solution	5% Minoxidil	Solution	Ipca Laboratories Ltd.	60
Brintop	5% Minoxidil	Solution	Brinton Pharmaceuticals Pvt. Ltd.	100
Anasure	5% Minoxidil	Solution	Sun Pharmaceutical Industries Ltd.	60
Minopep	5% Minoxidil	Solution	Indiabulls Pharmaceutical Ltd.	90
Mintop Yuva	5% Minoxidil	Solution	Dr. Reddy’s Laboratory Ltd.	60
Chymotra	5% Minoxidil	Solution	Adroit Life Science Pvt. Ltd.	60
Exidil	5% Minoxidil Solution	Solution	Sun Pharmaceutical Industries Ltd.	60
Minol	5% Minoxidil	Solution	Dermo Care Laboratories	60
Scino	5% Minoxidil	Solution	Encore Pharmaceuticals, Inc	60
Grewit	5% Minoxidil	Solution	Kivi Labs Ltd.	60
Tinfal	5% Minoxidil	Solution	Leeford Health Care	60
Regrow		Solution	West-coast Pharmaceuticals Works Ltd.	60
Minscalp		Solution	East West Pharma	60
Maxihair		Solution	Anhox Healthcare Pvt. Ltd.	60
Biominox		Solution	Merck Ltd.	100
Pilomin	5% Minoxidil	Solution	Palsons Derma	60
Gromo		Solution	Adcock Ingram Health Care Pvt. Ltd.	60
Pylodil	5% Minoxidil	Solution	Sol Derma Pharmaceuticals Pvt. Ltd.	60
Andromin		Solution	Med Manor Organics Pvt. Ltd.	100
Dividil Forte		Solution	Flagship Biotech International	60
Kazegrow	5% Minoxidil	Solution	Woakes Pharma	75
Hairbild	5% Minoxidil	Solution	Prosaic Pharmaceuticals Pvt. Ltd.	60
Hagain	5% Minoxidil	Solution	Dermcure’s Pharma	60
Primetop		Solution	Derma Prime	60
Stonark	5%	Solution	Cutis Dermacare	60
Minopen	5%	Solution	Pretium Pharmaceuticals	60
Minosilk	5% Minoxidil	Solution	Ethinnext Pharma	60
Nixdil Forte	5% Minoxidil	Solution	Biochemix Health Care Pvt. Ltd.	60
Pilogro Plus		Solution	Fulford India Ltd.	60
Hairise	5% Minoxidil	Solution	Carise Pharmaceuticals Pvt. Ltd.	60
Minovera	5% Minoxidil	Solution	Era Pharmaceuticals	60
Hairouse-MX		Solution	Nithyasha Health Care Pvt. Ltd.	60
Gzldil		Solution	Gedzrlvin Pharmaceuticals Pvt. Ltd.	60
Keshgaine		Solution	Connote Healthcare	60
Healing Pharma Rephair	5% Minoxidil	Solution	Healing Pharma Pvt. Ltd.	60
Biominox A		Solution	Sante Mernaud Pharmaceuticals Pvt. Ltd.	60
Stay Hairz	5% Minoxidil	Solution	Astra Labs	60
Mxd-5	5% Minoxidil	Solution	La Med India	60
Minofall	5% Minoxidil	Solution	Dr. Johns Laboratories Pvt. Ltd.	60
F Extend		Solution	Fluense Pharmaceuticals	60
Minoxidil	5%	Solution	Knoll Healthcare Pvt. Ltd.	60

### Men’s Rogaine 2%, 5% Aerosol (Easy to Use Foam)

Aerosol helps in hair growth when applied to the vertex part. Outline of this aerosol is described in Table 13 which includes ingredients that are used in this aerosol, type of dosage form, use of aerosol, what are storage conditions, and inactive ingredients used. Some of the drawbacks of this aerosol include; chest pain, rapid heartbeat, dizziness, increase in body weight, irritation on the scalp, undesired facial hair growth, no improvement after applying for 4 months.

**Table 13:** Men’s Rogaine 2%, 5% aerosol (easy to use foam)

Ingredients and dosage	Use	Types	Storage and direction of Use	Inactive ingredients
Minoxidil (w/w) without propellant 60 g can once a day for women baldness and twice a day for men pattern	Hair growth only vertex part	Aerosol, foam	20–25 °C Apply half a capful 2 times a day directly to the scalp in case of men and in case of women use once a day	Butane, BHT, cetyl alcohol, citric acid, glycerine, isobutane, lactic acid, polysorbate 60, propane, purified water, alcohol 40-B, stearyl alcohol

Precautions that one should be kept in mind include; only for external use; away for children; as it is in aerosol so it is extremely flammable.

### EquateTM

EquateTM which is Minoxidil 2%, 5% topical solution which contains alcohol, propylene glycol, purified water as inactive ingredients. It should be used only for the vertex portion; only for male baldness and applied two times a day directly to the scalp. It is contraindicated in the case of women’s baldness. As it contains alcohol so chances of fire are more; keep away for the flame as well as fire.

Some of the information about Minoxidil 2%, a 5% solution is given in CIMS. Information is elaborated in Table 14 that covers male and female adults, safety measures, contraindication as well as interaction and adverse drug reaction.

**Table 14:** Minoxidil 2%, 5% solution (CIMS data)

Male adult	Female adult	Safety alert	Contraindication	Interaction	ADR’s
2%, 5% Minoxidil solution, apply 1 mL to the scalp	2% Minoxidil solution, apply 1 mL to the scalp bid	Pheochromocytoma: patient with treated or untreated HTN, scalp abnormality (psoriasis, sunburn), shaved scalp	Patient with pulmonary HTN, Angina pectoris, chronic heart failure, renal impairment, pregnancy, and lactation, elderly, children	Additive effect with other hypotensive drugs, risk of orthostatic hypotension with sympathetic blocking drugs (like-Guanethidine), topical enhanced absorption with other topical medical preparation (corticosteroids, retinoids, occlusive ointment bases	Reflex techy, fluid retention, changes in ECG, Hypertrichosis, Pericarditis, Nausea, Headache, Polymenorrhoea, Allergic rashes, Stevens–Johnson syndrome
5% Minoxidil foam or aerosol, apply 1/2 capful to the scalp bid	5% Foam, aerosol, apply 1/2 capful to the scalp once a day				

### Inoxi 10% Solution

Inoxi 10% solution is commonly used for the treatment of hair growth and only male pattern baldness and effective treatment for hereditary hair loss; thinning of hair at the top of the scalp, not the front. This solution is manufactured by Psycoremedies which has 10% Minoxidil.

#### Mechanism of Action

Increasing blood circulation to hair follicles. That to leads more oxygen supply to hair follicles and leads to less death of hair scalp cells. During 1st 2 weeks of application, hair fall may increase temporarily this is a normal sign of medicine effectiveness.

Other available brands include; Morr 10% solution manufactured by Intas Pharmaceuticals; Grewit 10% solution manufactured by Kivi Labs Ltd.; Trichoton M 10% solution manufactured by Med Manor Organics Pvt. Ltd.

This solution is unsafe for women and children.

The Topical solution is available in 20 mg/60 mL which costs about Rs. 153.00 and 100 mg/60 mL which costs about Rs. 550.00.

### Inoxi Forte

Inoxi Forte which is 5% Minoxidil lotion manufactured by Psycoremedies and its mechanism of action includes widening blood vessels and opening potassium channel.

#### Uses

This lotion is used for the; Hair growth stimulation; Androgenic alopecia; Hereditary hair loss problems.

#### Supportive Measures

5% Minoxidil lotion has interaction with other drugs those include; Clonidine, Nadolol, Nicorandil, Propranolol, Tretinoin.

Side effects of this lotion comprise of increase in heart rate; chest pain; tenderness in the breast; rare palpitations.

The lotion is opposed in cases like hypersensitivity; angina pectrosis; pregnancy.

It must be stored at room temperature. Don’t freeze this lotion.

Apply only to scalp; avoid contact with eyes and mucous membrane.

### Minch

Minch containing about Minoxidil 3% and Minoxidil 12.5% (w/v) manufactured by Aamorb (Sioux) having volume 60 mL.

It is used to treat male pattern baldness only on the top of the scalp, not in front. It works by increasing the blood circulation, oxygen supply, and nutrition to the hair follicles. During the first 2 weeks of hair fall may enhance for a time that means medicine has shown effective results.

#### Expert Advice

Apply directly to the scalp, do not shampoo for 4 h after applying the medication, during first 2 weeks hair fall may increase.

#### Supportive Measures

Side effects include; irritation at the applied site; itching; headache.

#### Unsafe During Pregnancy

Other brands available for this include; more 3% solution by Intas Pharmaceuticals Ltd.; MNX-3 topical solution by Salve Pharmaceuticals Ltd.

If you have missed the dose take as soon as possible if it is too late then following the next day schedule but not double the dose.

### Radixil

Radixil is a topical solution of Minoxidil 2%, 5% and 10% manufactured by Signova Pharma Pvt. Ltd. Several side effects include; irregularity in a heartbeat; an increase in weight; shortness of breath; skin redness; irritation in the eye.

Different drug interactions with Radixil; Alprazolam; Corticosteroids; Guanethidine.

A substitute available includes Exidil 5%, Morr 5%, Rootz 5%, Tugain 5%.

### Pilogro

Pilogro is a topical 2%, 5% Minoxidil manufactured by Fulford. Its onset of action can be observed within 4–5 h after application and may cause sleepiness.

Side effects of this include; shortness of breath; chest pain; weight gain; an irregular heartbeat.

Contraindicated in patients with congestive heart failure, unsafe for pregnant and breastfeeding women.

### Regero

Regero, Minoxidil 2% topical solution manufactured by Shinto Organics Ltd. Having a volume of 60 mL. It is a type of vasodilator used in the treatment of male pattern baldness. Applied on the scalp directly not used for the front portion.

Side effects of this solution include; itching; headache; irritation; chest pain; unwanted hair growth to the undesired site; swollen hands and feet.

Other than these alternative brands are also available in Table 15.

**Table 15:** Alternative brand available for Regero

Brand name	Manufactured by
Mintop 2% solution	Dr. Reddy’s Laboratories
Tugain 2%	Cipla Ltd
Regaine 2%	Janssen Pharmaceuticals
MX2	Hedge and Hedge Pharmaceuticals
Black Crown 2%	Dermajoint India
Gainehair 2%	Wockhardt Ltd
Stonark 2%	Cutis Dermacare
Maxihair 2%	Anhox Health Care Pvt. Ltd
Brintop 2%	Brinton Pharmaceuticals Pvt. Ltd
Gzldil	Gedzrivin Pharma Pvt. Ltd
Hairdil 2%	Lyra Laboratories Pvt. Ltd

### Retreat

Retreat is Minoxidil 5% (w/w) topical gel manufactured by Segment Care whose volume is 60 g.

#### Mechanism of Action

Vasodilator expands blood vessels which promotes blood supply and provides nutrition and oxygen to hair follicles that prevent hair cell death and result in an improvement in hair growth by prolonging the action of chemical messenger anagen.

How to use: use it in the dose and duration as advised by the Doctor. Check the level for direction before using it. Clean and dry the affected area and then apply the gel. Wash hands after applying.

Some of the alternative brands include; Tugain 5% gel (Cipla Ltd.), Curlz pep gel 5% (w/v) (Canixa Life Science Pvt.), Regrowee 5% gel (w/w) (West Coast Pharmaceutical Works Ltd.), Menexil 5% gel (Dabur India Ltd.)

### Stonark

Minoxidil 5% (w/v) solution manufactured by Cutis Derma. Use it in the dose as prescribed by the physician. Alternative brands available for Stonark include; Tugain 5%-Cipla Ltd. Regaine 5%-Janssen Pharma. MX-5-Hedge and Hedge Pharmaceuticals, Mintop Forte 5%-Dr. Reddy’s Laboratories. Minoqilib 5%-Galderma India Pvt. Ltd.

### Ximinox

2%, 5% Minoxidil lotion manufactured by Rowan Biopharmaceuticals Pvt Ltd. Side effects of this lotion–irritation, itching, headache. Different alternative brands are available for lotion-Arodil lotion-Dr. Johns Laboratories Pvt. Ltd., AGA 1% lotion-Nidus Pharma Pvt. Ltd., Mxd 10 lotion-L Med India, Minfin 10% lotion-HBC Life Science Pvt. Ltd.

### Gaine Hair

Gaine hair is a 2% topical solution manufactured by Wockhardt Ltd. composed of Aminexil and Minoxidil. Other alternative brands available Tugain 2% Solution-Cipla Ltd., MX 2 Solution.

Hegde and Hegde Pharmaceuticals and Black Crown 2% Solution-Derma joint India.

Side effects of this solution headache, allergic reaction, rashes on the skin, and chest pain.

### Hair 4U

Hair 4U is lotion (60 mL) manufactured by Glenmark Pharmaceuticals which is composed of Min 2% w/v + diaminopyridine oxide 1.5% w/v, Min 5% w/diamino pyridine oxide 1.5% w/v, Min 10% w/diamino pyridine 1.5% w/v.

### Hair 4U 2% Lotion and Hair 4U Spray

Hair 4U 2% lotion manufactured by Glenmark Pharmaceuticals composed of Aminoxil and Minoxidil. Side effects of headache, skin rashes, edema, and dermatitis. It cannot be used in case of allergy.

Hair 4U Spray 60 mL manufactured by Glenmark Pharmaceutical Ltd. (Grace well) and composed of Minoxidil 2%, 5%, 10% + diamino pyridine oxide 1.5% Generic-Diamino pyridine oxide.

### Radixil A and Radixil F

Both the solutions are manufactured by Signova Pharma Pvt. Ltd. Composition of Radixil A includes Minoxidil 5% + diamino pyridine 1.5 and Radixil F includes Minoxidil 5% + 0.1% Finasteride. Side effects of this topical solution redness and dry skin change in hair color and texture. Keep away from children and must be stored at 15–25 °C.

### Stonark-AX and Stonark-2AX Solution

Both are topical solution manufactured by Cutis Derma Care. Composition of Stonark-AX Minoxidil 2% w/w + diaminopyridine oxide 1.5% w/w and Stonark-2AX Minoxidil 2% w/v + Aminexil 1.5% w/v and alternative available brands Hairjoy 2%-solution-Torrent Pharmaceuticals.

### Hairslim-F Topical Solution

Solution is lipid-based composed of Minoxidil 5% w/v + Finasteride 0.1% w/v. Adverse effects are dry skin, tingling, headache, dermatitis, erythema, dizziness. After applying to allow to dry for completely 2–4 h; hairdryer not to be used.

### Keshgaine-5%

Minoxidil 5% solution manufactured by Connote Health Care. It should be applied once or twice daily and the solution must contact the scalp for at least 4 h before washing.

### Regenepure Precision Minoxidil 5% Spray

Spray composed of deionized water, aloe vera gel, sodium-cocoyl isethionate, carbomer, niacin, PEG-8, hydrolyzed wheat protein, allantoin, ascorbic acid, Vit-B6, linolenic acid, ketoconazole, menthol, salicylic acid, PEG-25, lemon oil, ZnO, Polysorbate-80, Phenoxyethanol, FD, and C blue. For external use only.

### Formula 82F/M

Composed of 0.25% Finasteride + 5% Minoxidil, retinoic acid, oleanolic-acid, algae extract with propylene glycol. Must be used twice a day i.e. 30 drops. The common area that includes the crown, top of the scalp, and around hair transplants are the area that can be treated with this.

### Andro Block F

Formulation composed of Min 5% + Azelaic acid 12.5% + Finasteride 0.1% + Ketoconazole 2% which is manufactured by Empower Pharmacy. It should be stored at temperature 68–77°F and away from heat, moisture, and light.

### Minoxidil Store Plus

Formulation composed of Min 10% + Azelaic acid 5% + Finasteride 0.1%, ethyl alcohol, propylene glycol, applied to the scalp and avoided in patients having high blood pressure. Side effects are itching, difficulty in breathing, increase in heart rate.

### Regre Topical Spray

Spray composed of only 5% Minoxidil (60 mL), which is used twice a day.

### MX-2 Solution

The solution is composed of Minoxidil 2% manufactured by Hedge and Hedge Pharmaceuticals. This solution used only for male patterns. Other alternative available brands includes Mintop 2%, Tugain 2%, Black crown 2%, Regaine 2%.

### Coverit 5%

Minoxidil 5% belongs to Schedule H, Manufactured by Micro Labs Ltd. Side effects are skin redness, weight gain, headache, and irregular heart rate.

Warnings include during pregnancy, congestive heart failure, and breastfeeding and scalp irritation.

Drug–drug interaction includes Alprazolam-moderate, Corticosteroids-moderate, and Guanethidine-severe.

### Exidil 5% Solution

The solution comprises of Minoxidil 5% which is manufactured by Ranbaxy Laboratories Ltd.

### Rootz 5% Solution

A solution composed of Minoxidil 5% manufactured by Cipla Ltd.

### Tinfal

Composed of Minoxidil 5% (w/v), ethanol 95% and volume is 60 mL.

### Tinfal Plus

Composed of Minoxidil 5% + Aminexil 1.5%, volume 60 mL, and used on dry hair once at night time.

### Lipogaine (Men)

Serum composed of Minoxidil 5% with biotinyl-tripeptide, niacin, apple polyphenol. Not use more than 1 mL of serum each time, no massage required to apply directly to the scalp in the hair loss area. Stored at temperature 10–35 °C, away from children.

### Lipogaine (Women)

Composed of only Minoxidil 2% and apply 1 mL to the scalp in hair loss area, apply twice a day but at least 8 h after first applied.

Keep away from children as well as from fire.

### Imxia

Composed of Minoxidil 5% which is manufactured by KLM Laboratories Pvt. Ltd.

### Minokem

The spray contains Minoxidil 2%, 5% (60 mL) manufactured by Alkem Laboratories.

Severe interactions with Guanethidine and moderate with Alprazolam and Corticosteroids.

Associated side effects are weight gain, irritation in the eye, chest pain, and redness of the skin.

### Minotreat

Lotion that contains Minoxidil 5% manufactured by Ikon Remedies Pvt. Ltd.

For external use only and do not show interaction with any formulation.

#### Available Alternative Brands

Biodens hair lotion, Minotress lotion-Prism Life Science Ltd.., Arodil Forte lotion-Dr. Johns Laboratories Pvt. Ltd., TM 5 lotion-New trimed.

### Proanagen Solution

Composed of diaminopyrimidine oxide topical solution, diamino pyridine oxide 1.5% (w/v) in green apple skin extract base and Kopexil (1NCI name diaminopyrimidine oxide, trade name Aminexil) manufactured by Curatio Healthcare Pvt. Ltd.

Stored at a cool place, away from light, must be applied as instructions given by dermatologists.

#### Mechanism of Action of Aminexil

The mechanism of action of Aminexil and Minoxidil is only structurally related by conjecture. It prolongs the hair development phase through non-hormonal mechanisms. Furthermore, hair follicles get hardened by DHT are softened by Aminexil. It expands the blood vessels and provides better blood flow to the hair follicles. Commonly used in hair cosmetic products to fight against hair loss due to premature exhaustion of the hair root. The drug acts by stimulating or inhibiting a receptor or an enzyme or a protein most of the time. Medications are produced in such a way that the ingredients target the specific site and bring about chemical changes in the body that can stop or reverse the chemical reaction which is causing the disease.

#### Mechanism of Action of Diaminopyridine (3,4-Diaminopyridine)

Blocks calcium-dependent potassium channels and prolongs the duration of the action potential at motor nerve terminals; this enhances calcium influx into nerve endings and causes the release of acetylcholine [97]. It selectively blocks presynaptic fast voltage-gated potassium channels thus prolonging cell membrane depolarization and action potential, and increasing calcium transport into the nerve endings.

### Keraglo Eva

Keraglo Eva is Manufactured by Ipca Laboratories Ltd., generic uses include Biotin, Folic Acid, Selenium. Composed of Biotin (10 Mg), Folic Acid (300 Mcg), Selenium (40 Mcg) Gamma Linolenic Acid (found mostly in plant-based oils such as borrage seed oil), multivitamin, multimineral. Gamma-linolenic acid is an essential fatty acid that helps to stop hair loss, as well as the growth of hair. No side effects, have been reported at appropriate doses. Daily dosage is 1 tablet daily with a glass of water, before or after a meal.

#### Drug Interaction of Biotin

May Interact With Clozapine, Fluvoxamine, Haloperidol, Mexiletine, Imipramine, Olanzapine, Pentazocine, Propranolol, Tacrine, Theophylline, Zileuton, and Zolmitriptan. Carbamazepine, Phenytoin, Phenobarbital, Valproic Acid.

##### Drug Interaction of Folic acid

Fluorouracil, Sulphonamide, Phenytoin, Methotrexate, Sulfasalazine, Cholestyramine.

#### Side effects of Folic Acid

The common side effects are urge to vomit, bloating of stomach, excessive passage of wind, loss of appetite.

#### Drug Interaction of Selenium

Seborrheic dermatitis.

#### Side Effects of Selenium

Nausea, stomach upset, skin rash, acute toxicity.

### Biodens Hair Lotion

Hair lotion which is manufactured by Adonis Phytoceuticals Pvt. Ltd. and having a salt composition of Minoxidil 5% (w/v).

#### Side Effects of Biodens Lotion

Excessive hair growth on the face, Rash, Edema (swelling), Skin irritation.

#### Mechanism of Action

It works by increasing blood flow to the hair follicles on the scalp, which prevents hair cell death and also enhances new hair growth. It is effective for baldness or thinning at the top of the scalp but less effective at the front or for receding hairline. Biodens hair lotion is not appropriate for sudden or inexplicable hair loss. It is considered as a safe medicine to promote hair growth after hereditary hair loss (male pattern baldness).

Applied directly to the scalp area in the amount. Clean and dry your scalp before using it.

*Alternate Brands of lotion includes* Hairex Lotion-Swiss Pharma Pvt. Ltd.: Minotress Lotion-Prism Life Sciences Ltd.: TM 5 Scalp Lotion-Newtrimed.

## Formulations Under Clinical Trials [98]

Some the formulations are under clinical trials i.e. under the different phases of clinical trials that are elaborated in Table 16.

**Table 16:** Formulation under clinical trials

Composition	Clinical trial phase
Comparison between topical minoxidil 5% foam formulation and 2% minoxidil topical solution	Phase 3
5% Minoxidil topical solution when applied twice daily	Phase 3
5% Topical foam	Phase 2
Bimatoprost 1% formulation A and B	Phase 1
5% Minoxidil once a day vs 2% minoxidil topical solution	Phase 3
DA-4001C	Phase 1
5% Minoxidil	Phase 2
5% minoxidil + 5 mg finasteride + 200 mg spironolactone	Phase 4
Comparison between 3% minoxidil and solution + 0.1% finasteride lotion	Phase 3
Autologous human platelet lysate	Phase 1
2% Minoxidil or finasteride	Phase 2
DA-4001H DA-400L	Phase 1
Dietary supplement 400 mg/day	Not applicable
Low-level light therapy	Not applicable
Biological: PRP (platelet-rich plasma)	Not applicable
Pantovigar plus 2% + 2% minoxidil	Phase 2
Stem cell component extract	Not applicable
ENERGI-F701 (1 mL, twice/day)	Phase 2
Topical SM04554 solution	Phase 2
Topical vehicle solution	Phase 3
Hair stimulating complex (HSC)	Phase 1
Device: Dulbecco’s modified Eagle medium, DMEM	Phase 2
Botulinum toxin (injection)	Phase 2
Drug ATI-50002 topical solution	Phase 2
Topical SM04554 solution	Phase 2
X5 hair laser (device: sham)	Not applicable
LH80 PRO (device: sham)	Phase 2
Hair max laser device (control device)	Not applicable
FOL-005	Phase 2
Adipose-derived stem cells suspension injection	Phase 4
Tetrapeptide aldehyde proteasome inhibitor	Phase 2
PureGraft and celution system	Phase 2
Human autologous hair follicle cells	Phase 1 Phase 2
Drug: P‐3074	Phase 3
Conditioned media of umbilical cd blood‐derived stem cells	Not applicable
Adipose-derived stem cells suspension	Phase 4
Acupuncture	Not applicable
Erchonia MLS	Not applicable

## Conclusion

Topical minoxidil and finasteride can be useful adjuncts to hair transplant surgery for AGA. Topical use of this therapeutics leads less side effects than oral use. The oral finasteride causes different systemic side effects including liver metabolism, decreased sex drive, ejaculation disorder and so on. By using the topical formulations and other supplements we can easily reduce the side effects. Different natural supplements also helpful for preventing hair loss. According to patients medical history, it is always significant to be detailed and include over-the-counter vitamins, minerals, and supplements. It is very important to go for allergy history as some allergies may impede the use of certain CAMs. In this article we reviewed different topical marketed formulations of different brands, natural products, supplements which are reported to treat against androgenic alopecia.

## Abbreviations

AR: Androgenic receptor
DHT: Dihydrotestosterone
DNA: Deoxyribonucleic acid
PRP: Platelet-rich plasma
TrA: Triamcinolone acetonide
EGCG: Epigallocatechin-3-gallate
AGA: Androgenetic alopecia
NIH: National Institutes of Health
FDA: Food and Drug Administration
CAM: Complementary and alternative medicine
